# Efficient dissolved organic carbon production and export in the oligotrophic ocean

**DOI:** 10.1038/s41467-017-02227-3

**Published:** 2017-12-11

**Authors:** Saeed Roshan, Timothy DeVries

**Affiliations:** 1 Department of Geography, University of California, Santa Barbara, CA 93106 USA; 2 Earth Research Institute, University of California, Santa Barbara, CA 93106 USA

**Keywords:** Carbon cycle, Marine chemistry

## Abstract

Biologically fixed carbon is transferred from the surface to deep ocean as sinking particles or dissolved organic carbon (DOC). DOC is estimated to account for ~20% of global export production, but the degree to which this varies regionally has not been assessed at a global scale. Here we present the first observationally based global-scale assessment of DOC production and export, obtained by combining an artificial neural network estimate of the global DOC distribution, and a data-constrained ocean circulation model. Our results demonstrate that the efficiency of DOC production and export varies more than threefold across oceanographic regions. DOC production and export display a pronounced peak in the oligotrophic subtropical oceans, where DOC accounts for roughly half of the total organic carbon export. These stratified nutrient-depleted regions are expected to expand with future warming, amplifying the role of DOC in the biological pump, and magnifying the need to improve DOC cycling in climate models.

## Abstract (web summary)

The degree of regional variability in marine dissolved organic carbon (DOC) export production is poorly constrained on a global scale. Here, the authors combine an artificial neural network and a data-constrained ocean circulation model to show that the efficiency of DOC export varies 3-fold across regions.

## Introduction

Organic carbon produced by phytoplankton in the surface ocean is partitioned into a spectrum of sizes, each of which is exported to the subsurface ocean via different mechanisms. In contrast to gravity-controlled export of particulate organic carbon (POC), export of dissolved organic carbon (DOC) from the surface to the subsurface is controlled by mixing. The contribution of DOC to the export of biogenic carbon from the surface ocean has been estimated at ~20% globally^1,2^, and some studies suggest that DOC can account for more than 50% of the total carbon export on local to regional scales^3,4^. Mechanisms for regional differences in DOC export efficiency (the relative contribution of DOC to the total organic carbon export) are well documented and include variations in ambient nutrient concentrations and microbial community structure^5,6^. Nonetheless, the only basin-scale study of DOC production dynamics found that although the ratio of net DOC production to new production (as measured by the net uptake of nitrate, NO_3_^−^, in the surface ocean) can vary significantly with latitude, a constant ratio was sufficient to explain the surface DOC distribution of the Atlantic Ocean^7,8^. Since the ratio of C:N in sinking particles is also relatively constant^9^, it is not clear how the observed variability in DOC export efficiency arises. A global picture of DOC production and export would help to resolve this puzzle, but is currently lacking.

Here we develop a global picture of DOC production and export efficiency by applying an artificial neural network (ANN) to a global ocean DOC data set^10^. The ANN takes advantage of the fact that the DOC distribution in the ocean is controlled by the same physical and biological processes which also control well-sampled parameters such as salinity, temperature, macronutrients, chlorophyll, light penetration, and dissolved oxygen. These latter parameters are sufficiently highly sampled that global maps at 1° horizontal resolution have already been produced. By training an ANN using these global maps and the available DOC data, we are able to fill in gaps in the DOC observations and produce global maps of the DOC concentration at 1° horizontal resolution (see Methods). By coupling the ANN-mapped DOC concentrations to a global ocean circulation model, we are able to diagnose substantial regional variability in the efficiency of DOC production and export.

## Results

### Distribution of DOC

**Fig. 1:**
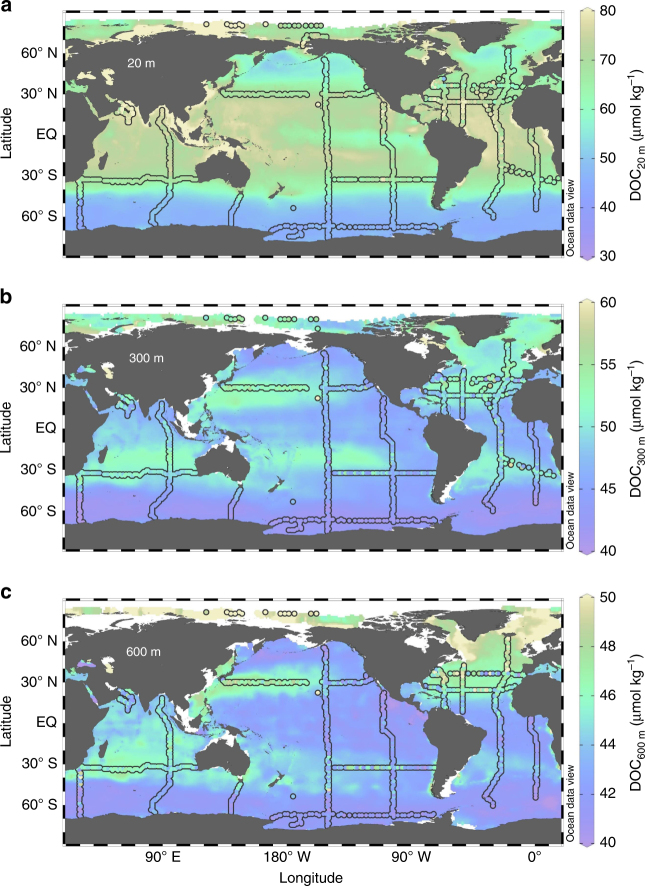
Distribution of ANN-derived and observational DOC. Color map is the artificial neural network (ANN)-derived dissolved organic carbon (DOC) concentration, and colored dots are the observed DOC concentration at 20 m (**a**), 300 m (**b**), and 600 m (**c**). ANN-derived DOC correlates with ~30,000 observed DOC data points with slope = 1.00, *R* ^2^ = 0.95, and RMSE = 2.37 μmol kg^−1^. A correlation study of data points in the upper 74 m indicates slope = 1.00, *R* ^2^ = 0.85, and RMSE = 4.36 μmol kg^−1^

The global surface DOC fields estimated by our ANN model (Fig. 1a) reveal the highest DOC concentrations in the tropics and subtropics of ~80 µmol kg^−1^. Lowest surface DOC concentrations of ~40 μmol kg^−1^ are found in the Southern Ocean, due to upwelling of DOC-depleted deep-ocean waters. At intermediate depths of 300–600 m, DOC concentrations are the highest in the North Atlantic, where deep-water formation brings DOC to a depth, and in the subtropical gyres where mode waters form(Fig. 1b, c).

### DOC production and export

To calculate the net DOC production in the surface ocean (hereafter abbreviated as NDP, also referred to as accumulation = [gross production − decomposition]), we used a diagnostic approach similar to the one previously applied to global observational fields of macronutrients^11,12^. In this approach, the ANN-produced DOC field is coupled to a data-constrained global ocean circulation model^13,14^, and the production of DOC in the surface ocean is simulated using a restoring model (see Methods section; ref. ^12^). We used the same method to calculate net NO_3_^−^ uptake in the surface ocean, which at steady state is equivalent to nitrate-fueled new production (see Methods). This method of calculating NDP and NO_3_^−^ uptake relies directly on the observed NO_3_^−^ and DOC fields, and circumvents the need to model DOC production and decomposition using parameterizations of food-web processes, as done in prognostic ocean biogeochemistry models (e.g., ref. ^15^). Our diagnostic approach can thus be used to test some of the assumptions regarding the relationship between new production and NDP that are built into prognostic climate models^16^. Since the export of DOC might take place at a different location from where it is produced, we also performed a separate simulation to diagnose net DOC export from the surface ocean (hereafter abbreviated as NDX; see Methods). One important limitation of this approach is that the majority of the DOC observations are made in summer months, when DOC tends to accumulate at the surface^3^, while our steady-state circulation model imposes winter-mixed layer depths in order to achieve proper ventilation of the deep ocean. Our approach thus assumes that all the DOC measured on the surface is preserved to be exported to the subsurface during winter, and thus has a lifetime of at least 3−6 months.

**Fig. 2:**
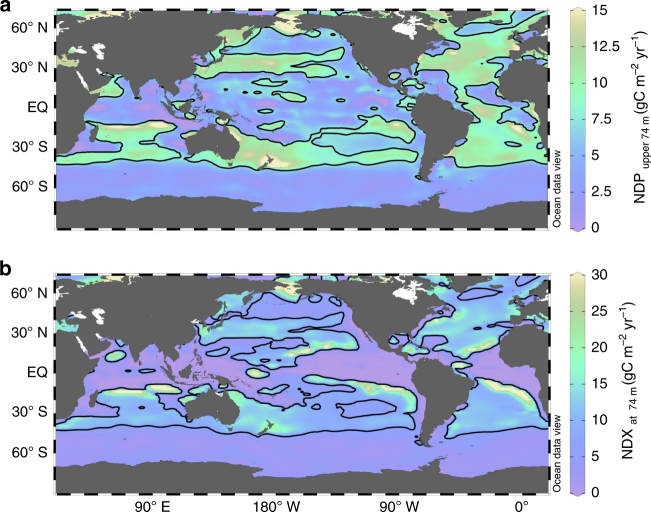
Maps of net DOC production and export fluxes. Net DOC production (NDP) in the upper 74 m (**a**) and net DOC export (NDX) below 74 m (**b**). At steady state, the global summation of NDX is equal to that of NDP, and is 2.31 ± 0.60 PgC yr^−1^

**Fig. 3:**
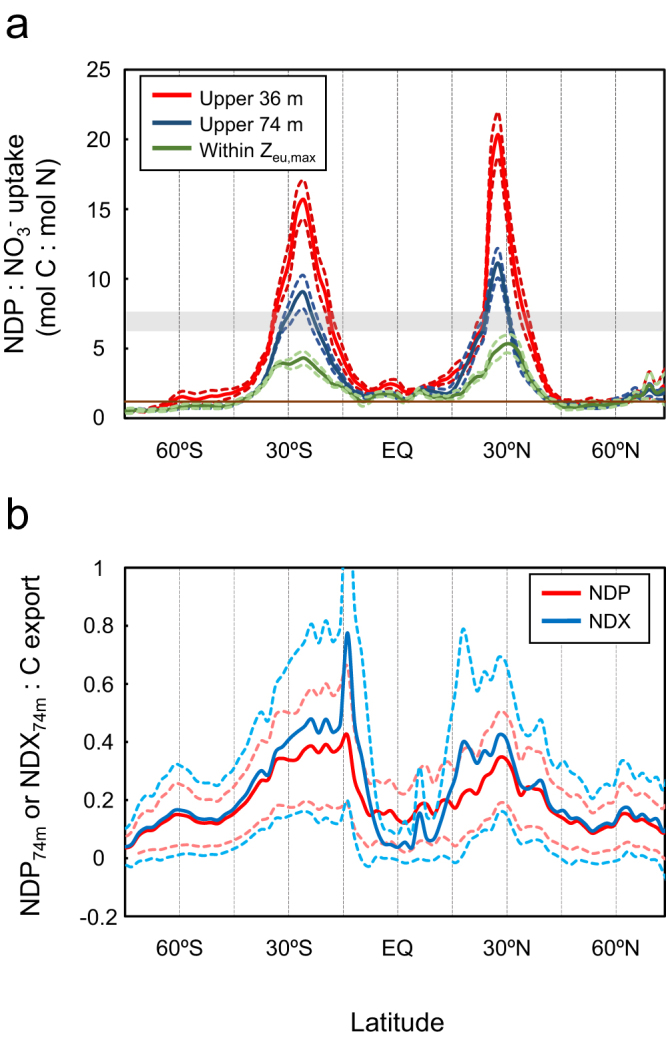
Latitudinal variability of DOC production and export to NO_3_^−^ uptake and C export. **a** Zonally averaged ratios of net DOC production (NDP) to NO_3_^−^ uptake within the upper 36 m (solid red line with dashed red lines indicating ±1 standard deviation), 74 m (solid blue line with dashed blue lines indicating ±1 standard deviation), and the deepest euphotic zone over the course of the year (*Z*_eu,max_; solid green line with dashed light green lines indicating ±1 standard deviation). Also shown is the global average ratio of C:N in POM (gray shading; ref. ^9^) and the mean NDP:NO_3_^−^ uptake ratio estimated in a previous study for the Atlantic Ocean (solid brown line; refs. ^7,8^). **b** Ratios of zonally averaged NDP (solid red line with dashed light red lines indicating ±1 standard deviation) and net DOC export (NDX; solid blue line with dashed light blue lines indicating ±1 standard deviation) to satellite-derived C export flux

Our results show that net DOC production differs from net DOC export at 74 m (the bottom of the second layer of our ocean model), but both show the largest values in the subtropical oceans, roughly between 20° and 40° (Fig. 2). Our diagnostic model finds that the global NDX to below 74 m (which is equal to global NDP within the upper 74 m at steady state) is 2.31 ± 0.60 PgC yr^−1^, similar to previous estimates^1,2,15^. However, the spatial variability of DOC production shows large deviations from that expected due to nutrient availability. The subtropical gyres are the regions with the lowest rates of net primary productivity (NPP) and POC export^17–19^. However, DOC production is as large as ~15 gC m^−2^ yr^−1^ in the subtropics (Fig. 2a), suggesting a decoupling of net DOC production from nutrient availability in these oligotrophic regions. Indeed, our diagnostic calculations of NDP and NO_3_^−^ uptake show large spatial variability across the ocean, with zonally averaged NDP:NO_3_^−^ uptake ratios (always in mol C:mol N if omitted hereafter) of >15:1 for production above 36 m in the subtropics (~30° N and ~30° S), and ~10:1 for production above 74 m (Fig. 3a). These ratios are even larger than the C:N ratios of exported particulate organic matter (POM), which is roughly constant throughout the ocean at about 6.3−7.7 mol C:mol N^9^. This suggests that DOC production significantly amplifies the total C production (the net community production) in the oligotrophic oceans.

Previous studies have shown that the ratio of carbon to nutrients in DOM is higher than the standard Redfield C:N ratio of ~6.6:1^20^. Prognostic models have used a C:N ratio ~11.8:1 for DOM^15^. Field studies also indicate a high C:N ratio of ~10 in DOM^21^. Our study offers the first global data-based assessment of the ratio between NDP and NO_3_^−^ uptake, and shows that the C:N ratio in DOM can vary widely between regions and vertically within the euphotic zone. The NDP:NO_3_^−^ uptake ratio is highest near the sea surface and decreases with depth in the euphotic zone. NDP:NO_3_^−^ uptake within the upper 36 m is ~15–20 in the subtropical gyres, and reaches a maximum of >30 in the subtropical South Pacific (Supplementary Fig. 1). The NDP:NO_3_^−^ uptake ratio is lower when averaged over the top 74 m, but still reaches values of ~7–10 in the subtropical gyres (Fig. 3a). Inasmuch as a portion of new NO_3_^−^ input is exported as POM, the C:N ratio in the DOM pool will be even higher than these NDP:NO_3_^−^ uptake ratios in the subtropics. Indeed, a recent observational study demonstrated that the C:N ratio of total organic matter was at least twice that of POM in the North Atlantic subtropical gyre^22^, suggesting that the C:N ratio of DOM is >13:1 in this region. In all, our results show that the nutrient efficiency of DOC accumulation in the upper ocean varies substantially with depth, region, and ocean basin (Supplementary Fig. 1). These results demonstrate that conditions such as temperature^23,24^, ambient nutrient concentration^6^, light availability^25,26^, or bacterial community composition^5^ might regulate the effect of new nutrients^7^ in fueling DOC accumulation.

### DOC contribution to total carbon export

To quantify the relative importance of DOC export to the biological pump, both globally and regionally, we compared our diagnosed net DOC production and export fluxes with C export fluxes derived from satellite measurements and empirical models (see Methods; refs. ^17–19^). The ratios of NDP and NDX to satellite-derived C export show a pattern similar to the diagnostically derived NDP:NO_3_^−^ uptake, with the highest values at subtropical latitudes (~15–30° N and S; Fig. 3b). In the subtropics, the ratio of NDP:C export is ~0.2–0.4, while in the equatorial region, this ratio is ~0.15, and in the high latitudes, it is ~0.1 or less (Fig. 3b). The latitudinal variation in NDP:C export is not quite as extreme as that of NDP:NO_3_^−^ uptake, which could reflect an additional source of new nutrients to the subtropical gyres from nitrogen fixation^27^. The pattern of NDX:C export is similar to that of NDP:C export, but export ratios are lower in the tropics and higher in the subtropics (Fig. 3b). This reflects the large-scale wind-driven circulation in the low latitudes: upwelling prevents DOC export near the equator, while poleward surface flows transport DOC accumulated in the tropics to the subtropics, where it is subducted. This sustains the balance between global DOC production and export at steady state (Supplementary Fig. 2). In the high latitudes, the production and export of DOC are nearly identical, reflecting the export of locally produced DOC (Fig. 3b). The globally-averaged ratio of DOC export to C export is 0.20 ± 0.09, which is in agreement with previous studies which suggest that DOC accounts for about 20% of organic matter export^1^. Our results are also in broad agreement with tracer-based estimates of total carbon export at two time-series study locations in the subtropical North Atlantic and subtropical North Pacific, both of which show a substantial contribution of DOC export total C export^4^.

## Discussion

**Fig. 4:**
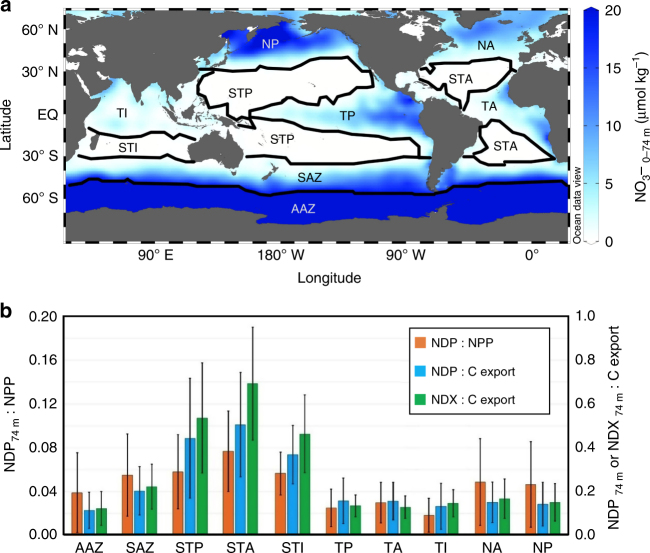
Regional variability of DOC production and export to NPP and C export. **a** Distribution of depth-integrated mean of nitrate above 74 m, which is used to regionalize the global ocean into distinct regions (solid borderlines): Antarctic Zone (AAZ), Sub-Antarctic Zone (SAZ), Sub-Tropical Pacific (STP), Atlantic (STA), and Indian (STI), and Tropical Pacific (TP), Atlantic (TA), and Indian (TI), and North Pacific (NP) and Atlantic (NA). **b** Ratio of regionally averaged NDP and NDX fluxes to satellite-derived net primary productivity (NPP) and C export fluxes for the regions shown in panel **a**. Error bars in **b** indicate ±1 standard deviation

What are the causes of the efficient DOC export in the subtropical gyres? One hypothesis that correlates well with the observations presented here is the inhibition of DOC consumption by bacteria under low-nutrient conditions^5,6^. To examine the correlation of NDP efficiency with nutrient availability, we partitioned the euphotic zone into 10 different regions based on their nutrient concentration and ocean basin (Fig. 4a; see Methods section). Averaging the NDP:C export ratio over the resulting regions reveals that the most oligotrophic ocean regions, the subtropical gyres which have NO_3_^−^ concentrations less than 0.5 μM, have NDP:C export ratios of ~0.35–0.5 (Fig. 4b). The ratio of DOC export:C export is even higher (~0.45–0.7) than NDP:C export in subtropical regions, reflecting the additional subduction of DOC produced in the tropics. Throughout most of the rest of the ocean, the NDP:C export ratio ranges from only 0.1 to 0.2 (Fig. 4b). Finally, the ratio of NDP:NPP ranges from 0.02 to 0.08, also attaining the highest values in the subtropical gyres (Fig. 4b).

**Fig. 5:**
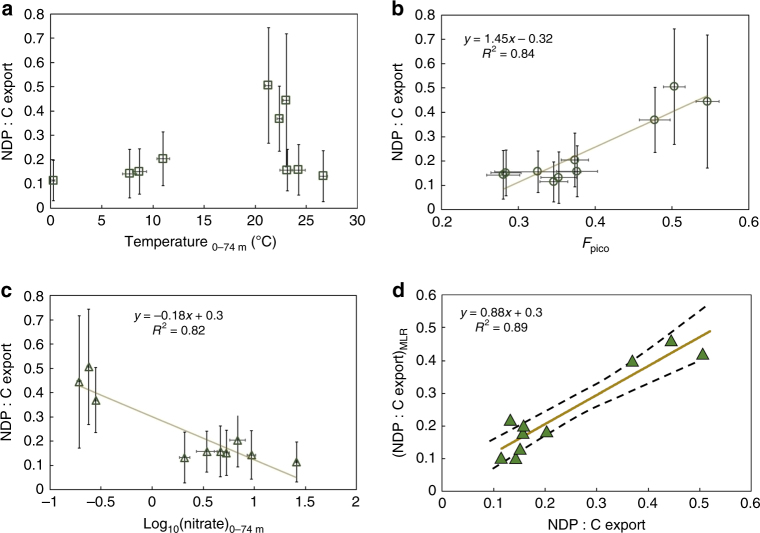
Regional relationship between DOC production:C export and several environmental parameters. Our diagnosed NDP:C export in the upper 74 m is plotted against regionally averaged temperature (**a**), *F*_pico_ (fraction of picoplankton) (**b**) and log_10_(nitrate) (**c**). The diagnosed NDP:C export ratios can be explained using a multilinear regression (MLR) model with two predictor variables (*F*_pico,_ and log_10_(nitrate)) (**d**). Error bars in **a**–**c** indicate ±1 standard deviation. Dashed envelope in **d** is the 95% confidence interval of the MLR prediction

Besides nutrient concentrations, other factors which can influence DOC production and export efficiency include temperature^23,24^ and ecosystem structure^5^. To investigate the effects of all of these factors on DOC export efficiency, we regressed the DOC production efficiency against the export-weighted mean temperature, NO_3_^−^, and fractional picoplankton abundance (*F*_pico_) averaged over the top 74 m within each of our 10 regions (Fig. 5). Here, picoplankton abundance stands as a proxy for ecosystem structure. Picoplankton are small plankton (<2 μm in diameter) which are abundant in nutrient-poor stably stratified ecosystems. Some picoplankton like *Prochlorococcus* have even lost the ability to utilize nitrate^28^, but remain efficient producers of many types of dissolved organic matter compounds^29^.

While previous studies have found increased partitioning of primary production to the DOC pool with increased temperature^23,24^, our regional analysis shows that net DOC production:C export does not increase monotonically as temperature increases, due to the fact that NDP:C export is low in tropical regions which have the highest sea-surface temperatures (Fig. 5a). On the other hand, our analysis shows a strong positive correlation of the NDP:C export ratio with *F*_pico_ (Fig. 5b), and a negative correlation between NDP:C export and NO_3_^−^ concentration (Fig. 5c). To combine the effect of all these parameters, we performed a multilinear regression (MLR) analysis using different combinations of these predictors. The MLR tests indicate that *F*_pico_ and log_10_(NO_3_^−^) are the most important predictors of NDP:C export ratio. Adding temperature as a predictor does not substantially improve the regression model (Supplementary Table 1). The derived regression equation NDP:C export = 0.710(±0.042) *F*_pico_ – 0.101(±0.021) log_10_(NO_3_^−^) (Supplementary Table 1) reproduces the regionally averaged NDP efficiency with an *R* ^2^ of 0.89 (Fig. 5d). The ratio of standardized coefficients of this MLR equation (i.e., $\frac{{\alpha F_{{\mathrm{pico}}}}}{{\alpha _{\log _{10}}({\mathrm{NO}}_{\mathrm{3}}^ - )}}$ = −1.17) indicates that the two predictors are almost equally important, but have opposite correlations with NDP:C export.

Although our diagnostic model does not reveal the mechanisms for the remarkably high DOC production efficiency in the oligotrophic subtropical ocean, high NDP efficiency appears to correlate significantly with ecosystem structure, as measured by the fractional picoplankton abundance. This suggests that ecological factors drive high DOC export from these regions. The stratified subtropical gyres are characterized by an efficient microbial loop, which processes NPP through the marine food web in the euphotic zone, building up a pool of more refractory DOC as fresh organic matter is degraded^30^. This refractory DOC only becomes available to marine microbes upon export out of the euphotic zone^3^. In contrast, the tropical and subpolar regions where nutrients are abundant are characterized by larger plankton communities, which promote the formation of fast-sinking particle aggregates and fecal pellets^31^ and a less-efficient food web. As well, high availability of nutrients in these regions facilitates the microbial consumption of DOC^6^. Finally, the substantial increase in DOC production efficiency in the upper water column (Fig. 3a) indicates that high light levels may also play an important role in stimulating DOC production^25,26^, and/or in inhibiting NO_3_^−^ uptake^28^.

The role of DOC export with respect to the surface bioassimilation of nutrients needs to be better considered in prognostic climate models^16^. Some of the current climate models are able to produce a pattern of surface DOC accumulation similar to that of observations^1^. However, a closer look at the ratio of simulated surface DOC to nutrient concentrations from these models shows a several-fold mismatch among the models, which are all significantly at odds with the observations (Supplementary Fig. 3). Our results highlight several mechanisms that could improve model estimates of carbon export, including tying DOC production and remineralization to light availability, nutrient concentration, and phytoplankton community structure.

The remarkably efficient production and export of DOC in the nutrient-depleted waters of the subtropical ocean suggest a vital role for DOC in the future ocean. As nutrient-poor regions expand with global warming and the associated stratification of surface waters^32–34^, efficient low-nutrient-demanding export of DOC from these regions will be an ever more important contributor to the oceanic biological sink of atmospheric CO_2_. Indeed, observations in the North Pacific subtropical gyre are already showing a long-term increase in surface DOC accumulation coincident with enhanced stratification^35^. Our analysis suggests that these trends will hold globally, amplifying the role of DOC in the ocean’s biological carbon sink in the future.

## Methods

### Observational data sets

The temperature, salinity, oxygen, and nutrient data were obtained from World Ocean Atlas 2013_v2 1° × 1° annual climatology (https://www.nodc.noaa.gov/OC5/woa13; refs. ^36–39^). Monthly SeaWiFS chlorophyll maps were downloaded from http://www.science.oregonstate.edu/ocean.productivity/index.php for the period of 1997–2009, and averaged to obtain a monthly climatology. Monthly SeaWIFS chlorophyll data were also used to calculate the depth of the euphotic zone according to the relationship of ref. ^40^. DOC data were obtained from a recent compilation^10^ plus CLIVAR repeat section A10 (completed in 2011) which was downloaded from http://cdiac.ornl.gov. Ocean bathymetric data were downloaded from http://www.gebco.net.

### ANN implementation

The global data set of DOC observations compiled by Letscher and Moore^10^ was binned into the World Ocean Atlas 2013_v2 1° × 1° grid (https://www.nodc.noaa.gov/OC5/woa13/). Approximately 30,000 grid points were represented by one or more observations. These DOC data points were taken as the target to train an ANN. Input data used for the ANN included nitrate, phosphate, apparent oxygen utilization, silicate, dissolved oxygen, salinity, temperature, potential density anomaly, depth, bottom depth, depth of the euphotic zone, and chlorophyll concentration, each provided on the same 1° × 1° grid as the DOC observations. In each training cycle, 70% of data were sampled and used for training and 30% were used for test and validation. We used a single hidden layer with 10–20 neurons fully connected, in a feed-forward architecture, to a single-node output layer. A sigmoid response function was used for the hidden layer, and a linear response function was used for the output layer. Levenberg–Marquardt and Bayesian regularization methods were used for back propagation. This architecture was determined to be the simplest ANN that could be trained to reproduce validation data sets with acceptable correlation metrics, and with no sign of overfitting.

An ensemble of 20 individual ANNs were trained by randomly sampling 70% of the data set, each of which were able to produce the validation subset (30% of withheld observations) with *R* ^2^ ≥ 0.95. Figure 1 shows the average of these 20 ANN-produced DOC fields. In addition to excluding 30% of the data during each training process for validation, we also excluded DOC data along CLIVAR A10 section during all the ANN training sessions. The correlation between the ANN-derived DOC and observational DOC along CLIVAR A10 section yields a slope, R^2^, and RMSE of 1.02, 0.87, and 3.47 μmol kg^–1^, respectively (Supplementary Fig. 4). Globally averaged profiles of mean difference and root-mean-square error (RMSE) between observed and ANN-derived DOC reveal almost no bias, with RMSE ranging from ~1 μmol kg^–1^ in the deep ocean to ~5 μmol kg^–1^ on the surface (Supplementary Fig. 5).

In a critical examination of the ANN approach, we excluded data from whole-ocean basins from our training subset, and examined the fit of the resulting model to data from the excluded basin. In these strong tests of the ANN approach, the ANN-predicted and observed DOC compare favorably in each basin (Supplementary Fig. 6). *R* ^2^ values for the ANN-predicted vs. observed DOC concentrations in the excluded basins vary from 0.8 (North Atlantic and Indian Ocean) to >0.9 (South Pacific and South Atlantic), indicating the robustness of the ANN approach. We should note that this test is especially strict for the Indian Ocean, since it contributes more than 30% of the total DOC observations.

### Diagnostic modeling

The ocean circulation model provides an off-line transport matrix that has been constrained by observational fields of salinity, potential temperature, natural ∆^14^C, CFC-11, sea-surface height anomaly, sea-surface heat, and freshwater fluxes^13,14^. After interpolation of the ANN-derived DOC and climatological nitrate fields from WOA13 grids (1° × 1° and 102 depth levels) to our ocean circulation model (2° × 2° and 24 depth levels), the following matrix equation that is a steady-state mass balance was solved:

1 $$\left[ {\begin{array}{*{20}{c}} {\begin{array}{*{20}{c}} {\frac{{{\mathrm{d}}{\mathbf{C}}_{\mathrm{s}}}}{{{\mathrm{d}}t}}} \end{array}} \\ {\frac{{{\mathrm{d}}{\mathbf{C}}_{\mathrm{i}}}}{{{\mathrm{d}}t}}} \end{array}} \right] = \left[ {\begin{array}{*{20}{c}} {{\mathbf{T}}_{{\mathrm{ss}}}} & {{\mathbf{T}}_{{\mathrm{si}}}} \\ {{\mathbf{T}}_{{\mathrm{is}}}} & {{\mathbf{T}}_{{\mathrm{ii}}}} \end{array}} \right] \left[ {\begin{array}{*{20}{c}} {{\mathbf{C}}_{\mathrm{s}}} \\ {{\mathbf{C}}_{\mathrm{i}}} \end{array}} \right] + \left[ {\begin{array}{*{20}{c}} {\mathbf{J}} \\ {\mathbf{L}} \end{array}} \right] = 0,$$

where **T**_ss_, **T**_si_, **T**_is_, and **T**_ii_ (units of yr^−1^) are the partitions of the transport matrix that circulate the tracer **C** (DOC or NO_3_^−^, units of µmol kg^−1^) from surface to surface, surface to interior, interior to surface, and interior to interior grid points, respectively. **C**_s_ and **C**_i_ are the tracer concentration at the surface and interior grid points, respectively. **J** and **L** are the net biological source or sink (i.e., production–decomposition for DOC, or uptake– regeneration for NO_3_^−^) of **C** on the surface and interior, respectively (units of µmol kg^−1^ yr^−1^). Since we seek only the **J** term, but not **L**, we can substitute the observed tracer concentration for **C**_i_ and write the first row of Eq. (1) separately, as

2 $${\mathbf{T}}_{{\mathrm{ss}}} {\mathbf{C}}_{\mathrm{s}} + {\mathbf{T}}_{{\mathrm{si}}} {\mathbf{C}}_{{\mathrm{i}},{\mathrm{obs}}} + {\mathbf{J}} = 0.$$

Here, **J** is the net uptake rate (for NO_3_^−^; **J**_up, nitrate_) or net production rate (for DOC; **J**_pr, DOC_; NDP rate) on the surface which is modeled by restoring to observations with a timescale *τ* (units of yr):

3 $${\mathbf{J}} = - {\mathbf{J}}_{{\mathrm{up}},{\mathrm{nitrate}}} = - \frac{{\left( {{\mathbf{C}}_{\mathrm{s}} - {\mathbf{C}}_{{\mathrm{s}},{\mathrm{obs}}}} \right)}}{\tau }{\mathrm{for}}\,\;{\mathrm{NO}}_{\mathrm{3}}^ - $$

4 $${\mathbf{J}} = {\mathbf{J}}_{{\mathrm{pr}},\,{\mathrm{DOC}}} = \frac{{\left( {{\mathbf{C}}_{{\mathrm{s}},{\mathrm{obs}}} - {\mathbf{C}}_{\mathrm{s}}} \right)}}{\tau }\,{\mathrm{for}}\,\;{\mathrm{DOC}}$$

**C**_s, obs_ and **C**_i, obs_ were set equal to the World Ocean Atlas climatology for NO_3_^−^ and to our ANN-derived climatology for DOC, and upon choosing a value of $\tau$ (see below), we solved Eqs. 2–4 for **C**_s_ and for **J**_up, nitrate_ and **J**_pr, DOC_ for each grid point in the surface ocean.

To investigate the depth variability of net DOC production within the euphotic zone (results shown in Fig. 3a and Supplementary Fig. 1), we ran three separate models using different depths to separate the surface and interior grid points. In the first model, we set the depth of the surface at the base of the first model layer (36 m). In the second model, we set the depth of the surface at the base of the second model layer (74 m). In the third model, we set the depth of the surface at the base of the model layer that is closest to (but not deeper than) the maximum depth of the euphotic zone. In this third model, the depth of the surface varies spatially due to variability in the depth of the euphotic zone. We emphasize that this model is entirely diagnostic, and does not rely on any assumptions regarding ecological or chemical processes in the upper ocean.

Wherever applicable, we used the density of seawater (from the equation of state of seawater using temperature, salinity, and pressure) to convert seawater mass-based units to seawater volume- or area-based units.

### Net DOC export calculation

To assess the net export of DOC (NDX) from the euphotic zone, we solved the equation

5 $$\frac{{{\mathrm{d}}{\mathbf{C}}}}{{{\mathrm{d}}t}} = {\mathbf{T}} {\mathbf{C}} + {\mathbf{J}}_{{\mathrm{pr}},{\mathrm{DOC}}} - k \cdot {\mathbf{C}} = 0$$

where **J**_pr, DOC_ is the rate of net production of DOC (NDP) derived from our diagnostic model (Eqs. 2 and 4), and *k* is set to zero everywhere except in the layer directly below the export depth (74 m in Figs. 2 and 3), where it is set to a very large value (10^12^ yr^–1^) in order to effectively remove all tracer (**C**) within the grid box directly below the export depth. Solving this equation for **C** at steady state yields the rate of export ($k \cdot {\mathbf{C}}$; in µmol kg^−1^ yr^−1^) needed to balance net DOC production above the export depth. The molar rate was converted to mass flux (gC m^−2^ yr^−1^) by applying the thickness of the third layer in the model, seawater density, and the atomic weight of carbon.

### Monte Carlo uncertainty assessment

The above equations were solved 2,000 times with random inputs of ANN-derived DOC field (i.e., one DOC field out of 20 possible DOC fields was chosen for each run), circulation matrix (10 different circulation matrices from ref. ^13^), and *τ* between 3 and 9 months. We note that using the 20 different DOC fields produced by the ANN in our model calculations allows us to propagate the spatially correlated errors of the ANN-predicted DOC fields. The results from these 2000 different models were taken to calculate averages and standard deviations of DOC production and NO_3_^−^ uptake. Uncertainty assessment of the export calculation was also performed in the same manner where the above production solutions and 10 different circulation matrices were used to solve Eq. 5.

### Satellite-based estimation of C export and *F*_pico_

Satellite-based C export was calculated using three empirical equations which require only temperature and NPP^17–19^. SeaWiFS NPP was downloaded from http://www.science.oregonstate.edu/ocean.productivity/index.php for all three NPP algorithms (VGPM^41^, Eppley-VGPM^42^, and CbPM^43^) and averaged over all months and years to produce an annual climatology. Overall, nine different satellite-derived C export maps were calculated using different combinations of NPP and export algorithm. These were averaged for Figs. 3b and 4b in the main text. *F*_pico_ was calculated from our derived annual NPP from 1997 to 2009 using a previously established relationship^31^.

### Regionalization

The regions in Fig. 4 were divided based on the depth-integrated mean of NO_3_^−^ concentration between 0 and 74 m. Subtropical regions in the Atlantic, Pacific, and Indian Oceans (STA, STP, and STI, respectively) were divided from tropical (TA, TP, and TI), northern (NA and NP), and southern (SAZ) regions at a mean NO_3_^−^ concentration of 0.5 μmol kg^–1^. We note that we used 0.5 μmol kg^−1^ nitrate concentration only as a convenient marker for the edge of the oligotrophic gyres in the annual nitrate climatology, and not to imply that this particular value of NO_3_^−^ results in nitrate-limited ecosystems. In the Southern Ocean, the Sub-Antarctic zone (SAZ) was divided from the Antarctic Zone (AAZ) at a mean NO_3_^−^ concentration of 20 μmol kg^−1^.

### CMIP5 result analysis

The results of CMIP5 models GFDL-ESM2M^44^, CNRM-CM5^45^, IPSL-CM5A-LR^46^, and CESM1-BGC^47^ were downloaded from http://pcmdi9.llnl.gov. Annual data sets of DOC and NO_3_^−^ from historical simulations were averaged over the time period 1956–2005. The resulting DOC and NO_3_^−^ fields were then averaged vertically for the upper 100 m, and then zonally. The ratios of the final average for DOC and NO_3_^−^ were then calculated and plotted in Supplementary Fig. 3. Before the ratio calculation, 38 μmol kg^−1^ DOC (the concentration of refractory DOC observed in the deep North Pacific Ocean) was added to the zonal average of DOC for the CNRM-CM5, IPSL-CM5A-LR, and CESM1-BGC models, which do not include a refractory DOC pool^1^.

### Map visualization

Color maps in Figs. 1, 2, and 4 were produced using Ocean Data View 4.7.7 software^48^. The gridding was performed through weighted averaging applying 15‰ for both *X* and *Y* axes scale-lengths.

### Data availability

The data produced in this study are available from the corresponding author upon request.

## Electronic supplementary material

Supplementary Information

## References

[1] Hansell, D. A., & Carlson, C. A. (eds). *Biogeochemistry of Marine Dissolved Organic Matter* (Academic Press, London, 2014).

[2] Hansell DA, Carlson CA, Repeta DJ, Schlitzer R (2009). Dissolved organic matter in the ocean: a controversy stimulates new insights. Oceanography.

[3] Carlson CA, Ducklow HW, Michaels AF (1994). Annual flux of dissolved organic carbon from the euphotic zone in the northwestern Sargasso Sea. Nature.

[4] Emerson S (2014). Annual net community production and the biological carbon flux in the ocean. Glob. Biogeochem. Cycles.

[5] Carlson CA (2002). Effect of nutrient amendments on bacterioplankton production, community structure, and DOC utilization in the northwestern Sargasso Sea. Aquat. Microb. Ecol..

[6] Letscher RT (2015). Microbial community composition and nitrogen availability influence DOC remineralization in the South Pacific Gyre. Mar. Chem..

[7] Romera-Castillo C, Letscher RT, Hansell DA (2016). New nutrients exert fundamental control on dissolved organic carbon accumulation in the surface Atlantic Ocean. Proc. Natl Acad. Sci. USA.

[8] Stubbins A (2016). A carbon for every nitrogen. Proc. Natl Acad. Sci. USA.

[9] Martiny AC (2013). Strong latitudinal patterns in the elemental ratios of marine plankton and organic matter. Nat. Geosci..

[10] Letscher RT, Moore JK (2015). Preferential remineralization of dissolved organic phosphorus and non‐Redfield DOM dynamics in the global ocean: Impacts on marine productivity, nitrogen fixation, and carbon export. Glob. Biogeochem. Cycles.

[11] DeVries T, Deutsch C (2014). Large-scale variations in the stoichiometry of marine organic matter respiration. Nat. Geosci..

[12] Weber T, Cram JA, Leung SW, DeVries T, Deutsch C (2016). Deep ocean nutrients imply large latitudinal variation in particle transfer efficiency. Proc. Natl Acad. Sci. USA.

[13] DeVries T, Primeau F (2011). Dynamically and observationally constrained estimates of water-mass distributions and ages in the global ocean. J. Phys. Oceanogr..

[14] DeVries T (2014). The oceanic anthropogenic CO_2_ sink: storage, air‐sea fluxes, and transports over the industrial era. Glob. Biogeochem. Cycles.

[15] Letscher RT, Moore JK, Teng YC, Primeau F (2015). Variable C: N: P stoichiometry of dissolved organic matter cycling in the Community Earth System Model. Biogeosciences.

[16] Taylor KE, Stouffer RJ, Meehl GA (2012). An overview of CMIP5 and the experiment design. Bull. Am. Meteorol. Soc..

[17] Dunne JP, Armstrong RA, Gnanadesikan A, Sarmiento J (2005). Empirical and mechanistic models for the particle export ratio. Glob. Biogeochem. Cycles.

[18] Laws EA, D’Sa E, Puneeta N (2011). Simple equations to estimate ratios of new or export production to total production from satellite-derived estimates of sea surface temperature and primary production. Limnol. Ocean. Methods.

[19] Laws EA, Falkowski PG, Smith WO, Ducklow H, McCarthy JJ (2000). Temperature effects on export production in the open ocean. Glob. Biogeochem. Cycles.

[20] Redfield AC (1958). The biological control of chemical factors in the environment. Am. Sci..

[21] Hopkinson CS, Vallino JJ (2005). Efficient export of carbon to the deep ocean through dissolved organic matter. Nature.

[22] Singh A, Baer SE, Riebesell U, Martiny AC, Lomas MW (2015). C: N: P stoichiometry at the Bermuda Atlantic Time-series Study station in the North Atlantic Ocean. Biogeosciences.

[23] Wohlers J (2009). Changes in biogenic carbon flow in response to sea surface warming. Proc. Natl Acad. Sci. USA.

[24] Kim JM (2011). Shifts in biogenic carbon flow from particulate to dissolved forms under high carbon dioxide and warm ocean conditions. Geophys. Res. Lett..

[25] Mueller B, den Haan J, Visser PM, Vermeji MJA, van Duyl FC (2016). Effect of light and nutrient availability on the release of dissolved organic carbon (DOC) by Caribbean turf algae. Sci. Rep..

[26] Cherrier J, Valentine S, Hamill B, Jeffrey WH, Marra JF (2015). Light-mediated release of dissolved organic carbon by phytoplankton. J. Mar. Syst..

[27] Karl D (1997). The role of nitrogen fixation in biogeochemical cycling in the subtropical North Pacific Ocean. Nature.

[28] Bragg JG, Dutkiewicz S, Jahn O, Follows MJ, Chisholm SW (2010). Modeling selective pressures on phytoplankton in the Global Ocean. PLoS ONE.

[29] Zhao Z (2017). Picocyanobacteria and deep-ocean fluorescent dissolved organic matter share similar optical properties. Nat. Commun..

[30] Jiao N (2010). Microbial production of recalcitrant dissolved organic matter: long-term carbon storage in the global ocean. Nat. Rev. Microbiol..

[31] Hirata T (2011). Synoptic relationships between surface Chlorophyll-a and diagnostic pigments specific to phytoplankton functional types. Biogeosciences.

[32] Bopp L (2013). Multiple stressors of ocean ecosystems in the 21st century: projections with CMIP5 models. Biogeosciences.

[33] Sarmiento JL (2004). Response of ocean ecosystems to climate warming. Glob. Biogeochem. Cycles.

[34] Behrenfeld MJ (2006). Climate-driven trends in contemporary ocean productivity. Nature.

[35] Church MJ, Ducklow HW, Karl DM (2002). Multiyear increases in dissolved organic matter inventories at Station ALOHA in the North Pacific Subtropical Gyre. Limnol. Oceanogr..

[36] Locarnini, R. A. et al. in *World Ocean Atlas 2013, Volume 1: Temperature* (ed. Levitus, S.) (A. Mishonov Technical Ed.; NOAA Atlas NESDIS 73, US Gov Print Off, 2013).

[37] Zweng, M. M. et al. in *World Ocean Atlas 2013, Volume 2: Salinity* (ed. Levitus, S.) (A. Mishonov Technical Ed.; NOAA Atlas NESDIS 74, US Gov Print Off, 2013).

[38] Garcia, H. E. et al. in *World Ocean Atlas 2013, Volume 3: Dissolved Oxygen, Apparent Oxygen Utilization, and Oxygen Saturation* (ed. Levitus, S.) (A. Mishonov Technical Ed.; NOAA Atlas NESDIS 75, US Gov Print Off, 2014).

[39] Garcia, H. E. et al. in *World Ocean Atlas2013, Volume 4: Dissolved Inorganic Nutrients* (phosphate, nitrate, silicate) (ed. Levitus, S.) (A. Mishonov Technical Ed.; NOAA Atlas NESDIS 76, US Gov Print Off, 2014).

[40] Morel A (2007). Examining the consistency of products derived from various ocean color sensors in open ocean (Case 1) waters in the perspective of a multi-sensor approach. Remote Sens. Environ..

[41] Behrenfeld MJ, Falkowski PG (1997). Photosynthetic rates derived from satellite‐based chlorophyll concentration. Limnol. Oceanogr..

[42] Carr ME (2006). A comparison of global estimates of marine primary production from ocean color. Deep-Sea Res. II.

[43] Behrenfeld MJ, Boss E, Siegel DA, Shea DM (2005). Carbon‐based ocean productivity and phytoplankton physiology from space. Glob. Biogeochem. Cycles.

[44] Dunne JP (2012). GFDL’s ESM2 global coupled climate-carbon earth system models. Part I: Physical formulation and baseline simulation characteristics. J. Clim..

[45] Voldoire A (2013). The CNRM-CM5.1 global climate model: description and basin evaluation. Clim. Dyn..

[46] Dufresne JL (2013). Climate change projections using the IPSL-CM5 EarthSystem Model: from CMIP3 to CMIP5. Clim. Dyn..

[47] Hurrell JW (2013). The community earth system model: a framework forcollaborative research. Bull. Am. Meteor. Soc..

[48] Schlitzer, R. Ocean Data View http://odv.awi.de (2015).

