# Author Correction: Efficient dissolved organic carbon production and export in the oligotrophic ocean

**DOI:** 10.1038/s41467-021-24980-2

**Published:** 2021-09-06

**Authors:** Saeed Roshan, Timothy DeVries

**Affiliations:** 1 Department of Geography, University of California, Santa Barbara, CA 93106 USA; 2 Earth Research Institute, University of California, Santa Barbara, CA 93106 USA

Correction to: *Nature Communications* 10.1038/s41467-017-02227-3, published online 11 December 2017.

The original version of this Article contained an error in the horizontal axes labelling of Fig. 3, in which the southernmost coordinates were labelled “N” instead of “S”. This has been corrected in both the PDF and HTML versions of the Article.

