# Supplementary material for: Efficient dissolved organic carbon production and export in the oligotrophic ocean
Source: Nat Commun. 2017 Dec 11;8:2036. doi: 10.1038/s41467-017-02227-3 (PMC5725454; doi:10.1038/s41467-017-02227-3)
Supplement: Supplementary file 1 — Supplementary Information [file 41467_2017_2227_MOESM1_ESM.pdf]

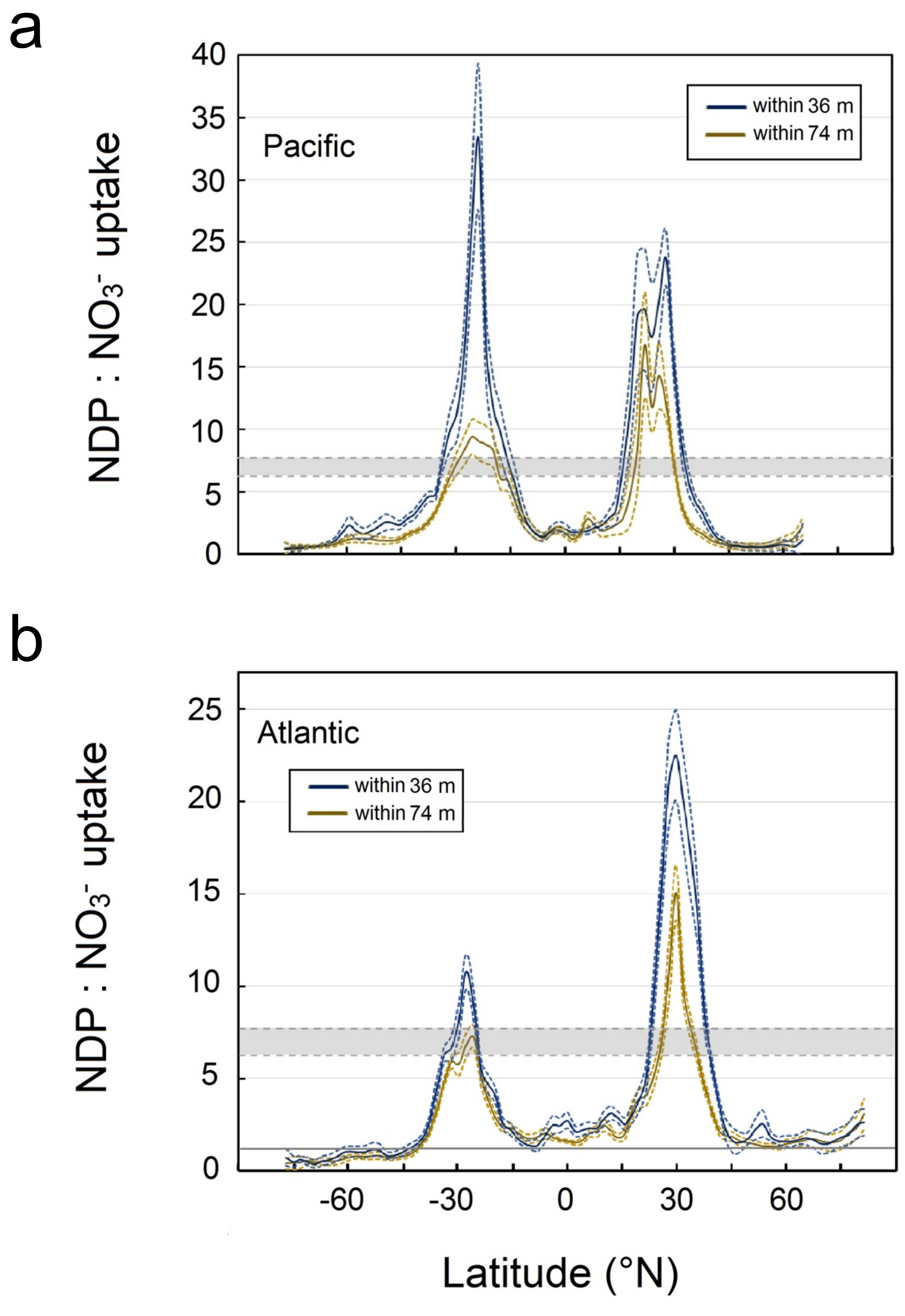

Supplementary Figure 1 | Ratio of net DOC production (NDP) to nitrate uptake in different ocean basins. Zonally-averaged ratio for the Pacific Ocean (**a**) and Atlantic Ocean (**b**).

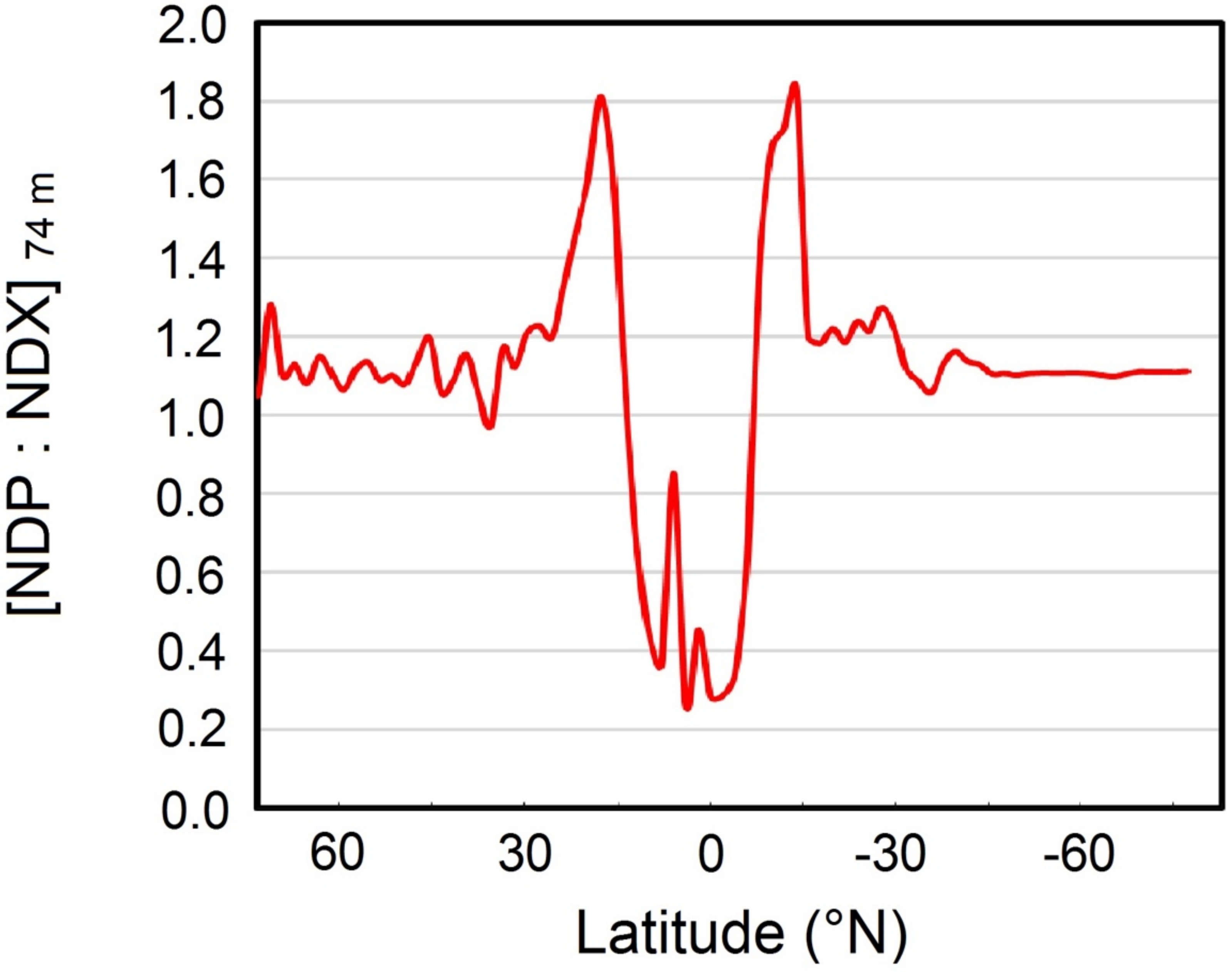

Supplementary Figure 2 | Ratio of zonally-averaged DOC export at 74 m depth to DOC production above 74 m.

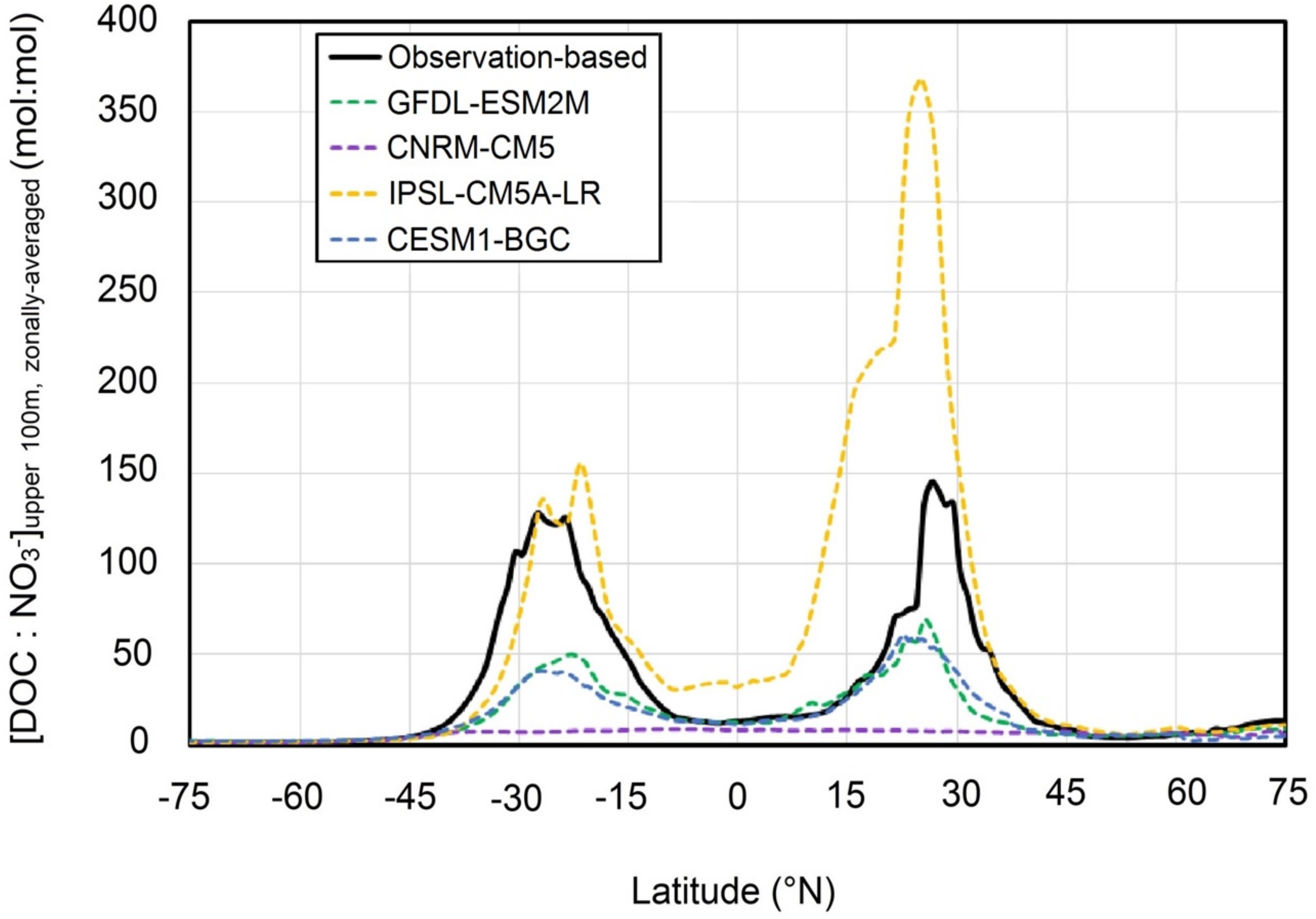

Supplementary Figure 3 | Ratio of zonally-averaged concentrations of DOC and NO_3^- in the upper 100 m from our observation-based ANN model (for DOC) and the World Ocean Atlas database (for NO_3^-), and the corresponding ratios simulated by CMIP5 models.

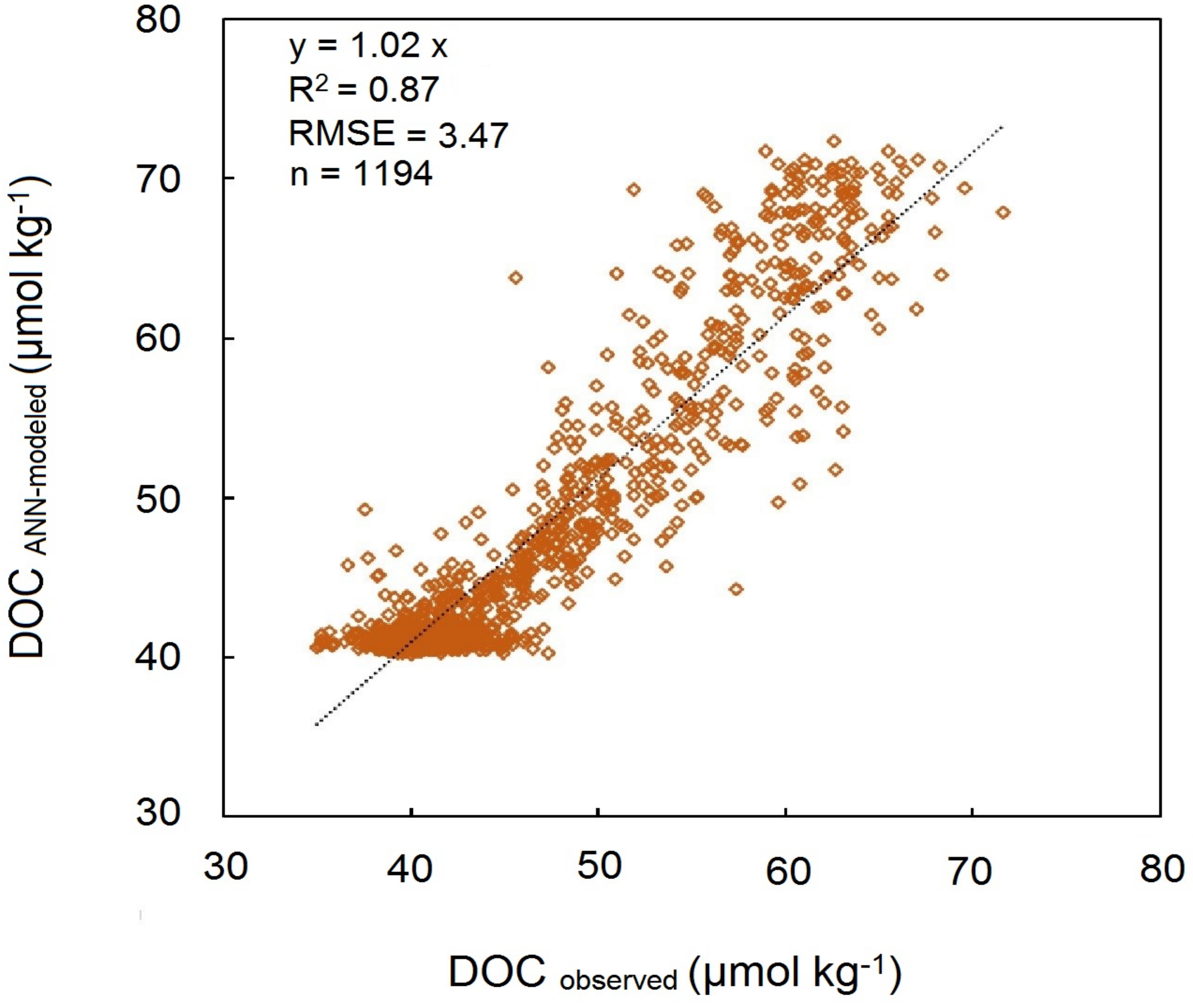

Supplementary Figure 4 | ANN-derived DOC vs. observed DOC along CLIVAR repeat section A10.

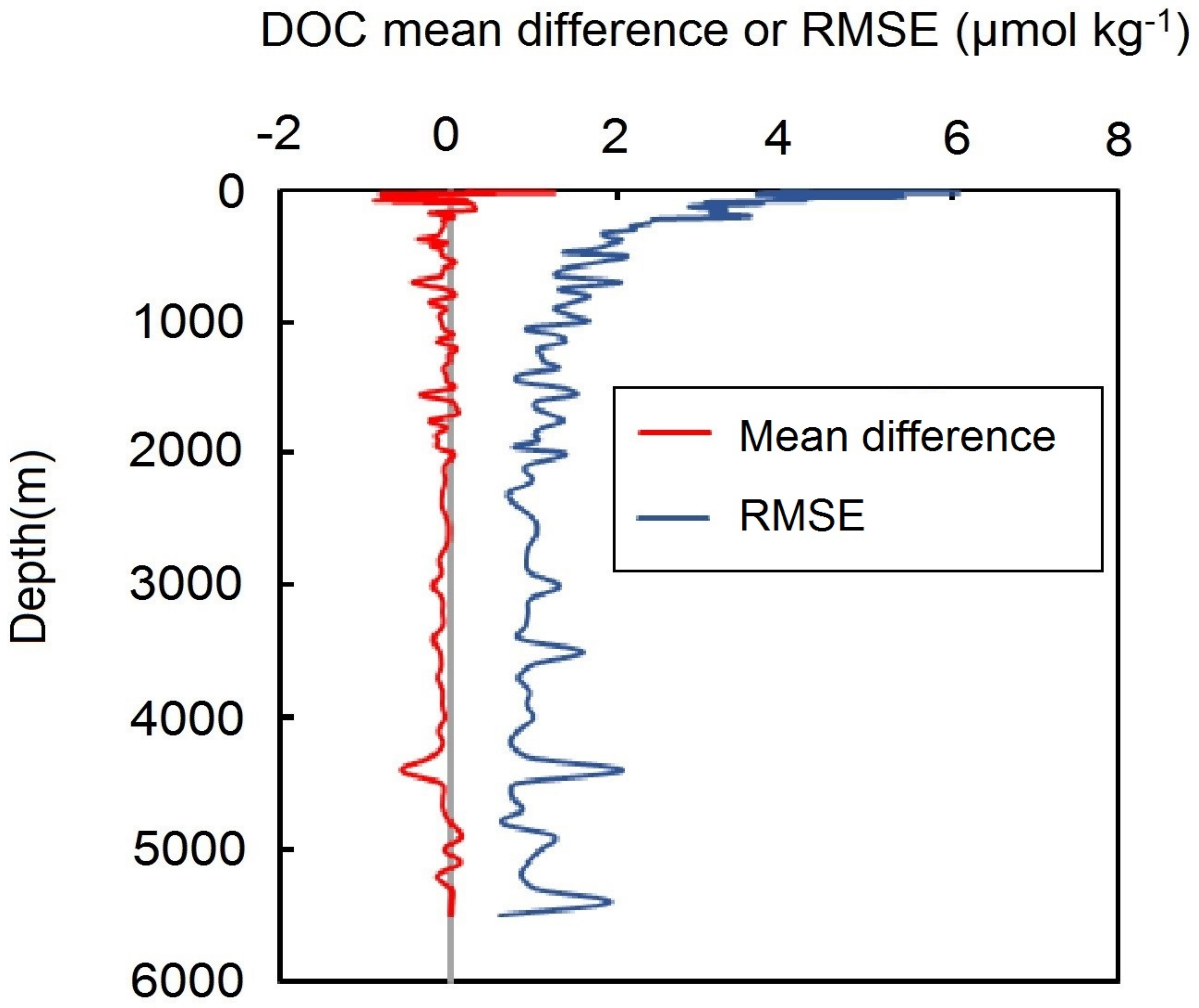

Supplementary Figure 5 | The layer-averaged mean difference and RMSE between observed and ANN-derived DOC.

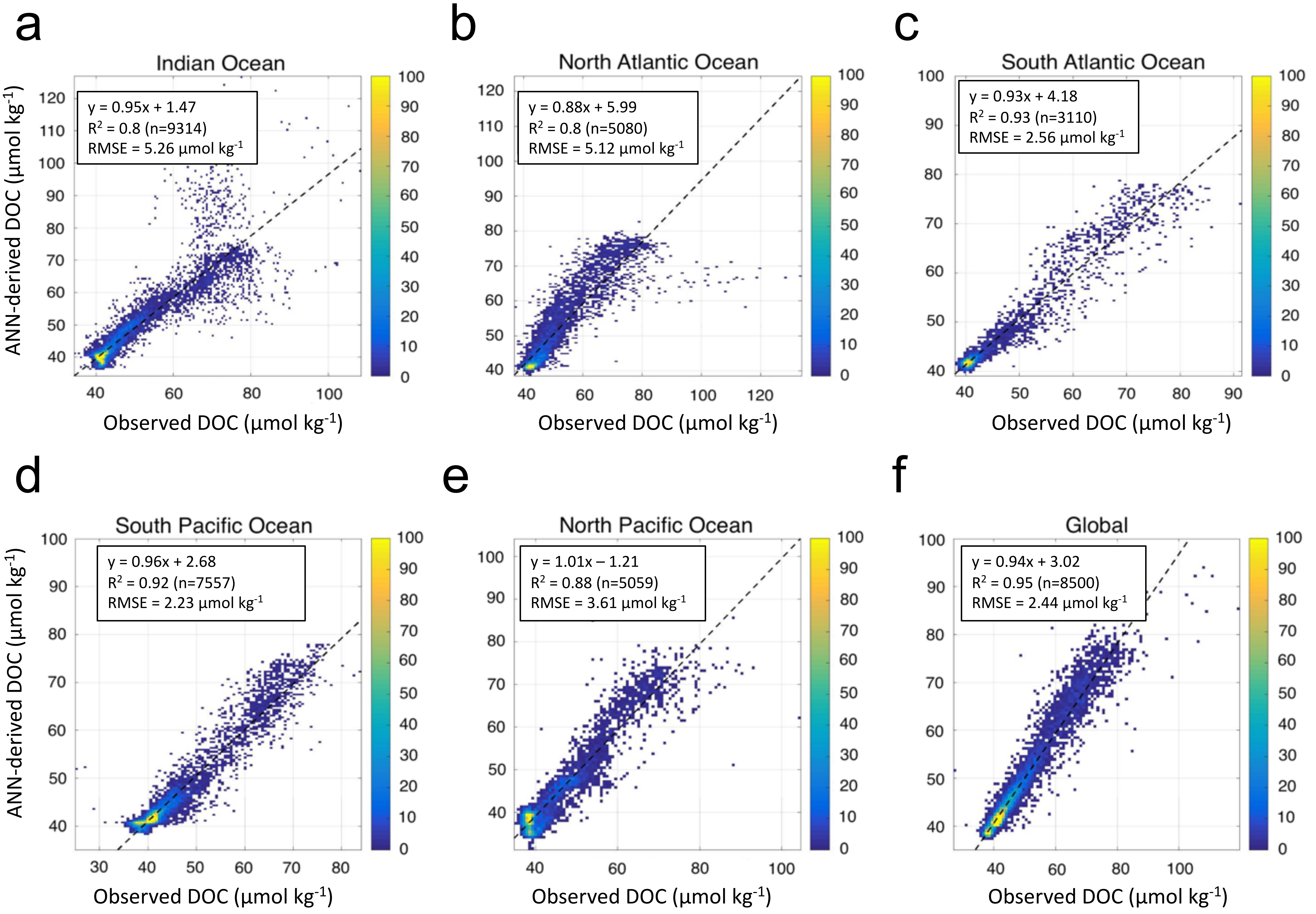

Supplementary Figure 6 | ANN-derived DOC vs. observed DOC for regionally-trained ANNs. In each of the plots **a**-**e**, observations from that region were excluded from the training dataset of our ANN model, and the resulting ANN-predicted DOC is compared against the observed DOC in that region. The good fit to observations even in regions that were excluded from the training dataset indicates the robustness of the ANN method. Panel **f** shows results from one of the ANN models used in our analysis, in which the validation dataset consists of a randomly selected subset (30%) of the global DOC observations.

Supplementary Table 1 | Analysis of MLR models to predict NDP flux : C export flux.

#Predictors	1		2		3	
Predictors	Coefficient(s)	p-value	Coefficient(s)	p-value	Coefficient(s)	p-value
F_{pico}	0.655	1.1 E-05	0.710	1.59 E-07	0.906	2.02 E-05
$log_{10}(NO_3^-)$			-0.101	1.41 E-03	-0.116	3.55 E-04
T					-0.0044	0.0495
	Adjusted R^2		Adjusted R^2		Adjusted R^2	
	0.783		0.844		0.837	
